# Autophagy: novel applications of nonsteroidal anti‐inflammatory drugs for primary cancer

**DOI:** 10.1002/cam4.1287

**Published:** 2017-12-28

**Authors:** Chen Yu, Wei‐bing Li, Jun‐bao Liu, Jian‐wei Lu, Ji‐feng Feng

**Affiliations:** ^1^ Department of Integrated TCM & Western Medicine Jiangsu Cancer Hospital Jiangsu Institute of Cancer Research Nanjing Medical University Affiliated Cancer Hospital Nanjing Jiang Su 210000 China; ^2^ Department of Traditional Chinese Medicine Henan Provincial People's Hospital Zhengzhou Henan China; ^3^ Department of Medicine Jiangsu Cancer Hospital Jiangsu Institute of Cancer Research Nanjing Medical University Affiliated Cancer Hospital Nanjing Jiang Su 210000 China

**Keywords:** Autophagy, nonsteroidal anti‐inflammatory drugs, programmed cell death

## Abstract

In eukaryotic cells, autophagy is a process associated with programmed cell death. During this process, cytoplasmic proteins and organelles are engulfed by double‐membrane autophagosomes, which then fuse with lysosomes to form autolysosomes. These autolysosomes then degrade their contents to recycle the cellular components. Autophagy has been implicated in a wide variety of physiological and pathological processes that are closely related to tumorigenesis. In recent years, an increasing number of studies have indicated that nonsteroidal anti‐inflammatory drugs, such as celecoxib, meloxicam, sulindac, aspirin, sildenafil, rofecoxib, and sodium salicylate, have diverse effects in cancer that are mediated by the autophagy pathway. These nonsteroidal anti‐inflammatory drugs can modulate tumor autophagy through the PI3K/Akt/mTOR, MAPK/ERK1/2, P53/DRAM, AMPK/mTOR, Bip/GRP78, CHOP/ GADD153, and HGF/MET signaling pathways and inhibit lysosome function, leading to p53‐dependent G1 cell‐cycle arrest. In this review, we summarize the research progress in autophagy induced by nonsteroidal anti‐inflammatory drugs and the molecular mechanisms of autophagy in cancer cells to provide a reference for the potential benefits of nonsteroidal anti‐inflammatory drugs in cancer chemotherapy.

## Background

Over the past few decades, much of cancer research has been focused on the mechanisms of apoptosis. However, apoptotic resistance has become a major obstacle in cancer treatment. Thus, studying the other mechanisms associated with programmed cell death has become increasingly important. Based on their associated morphological features, programmed cell death can be divided into apoptosis, autophagy, necrosis, and mitotic catastrophe. Recent studies have shown that changes in the external or internal environments (i.e., amino acid deficiency, insufficient glucose supply, reduced oxygen supply, and mitochondrial damage) can induce autophagy 1. Based on the substrate for each enzyme, autophagy can be subdivided into different pathways, including microautophagy, great‐autophagy, and molecular chaperone‐mediated autophagy.

Autophagy is a conserved catabolic process, and over the past decade, multiple studies have reported genetic and functional links between impaired autophagy and cancer, suggesting that autophagy is a mechanism of tumor suppression. During autophagy, cellular contents are enclosed within autophagosomes, which fuse with lysosomes to degrade and recycle their contents 2. In tumorigenesis, the function of autophagy is complex, since it is not only a prodeath mechanism but also a survival strategy under cellular stress 3, 4. The prodeath or prosurvival nature of the response may be related to tumor type, stage, and the ability of the tumor cells to sustain themselves. Some studies have reported that inhibition of autophagy enhances cellular apoptosis 5, 6.

**Figure 1:**
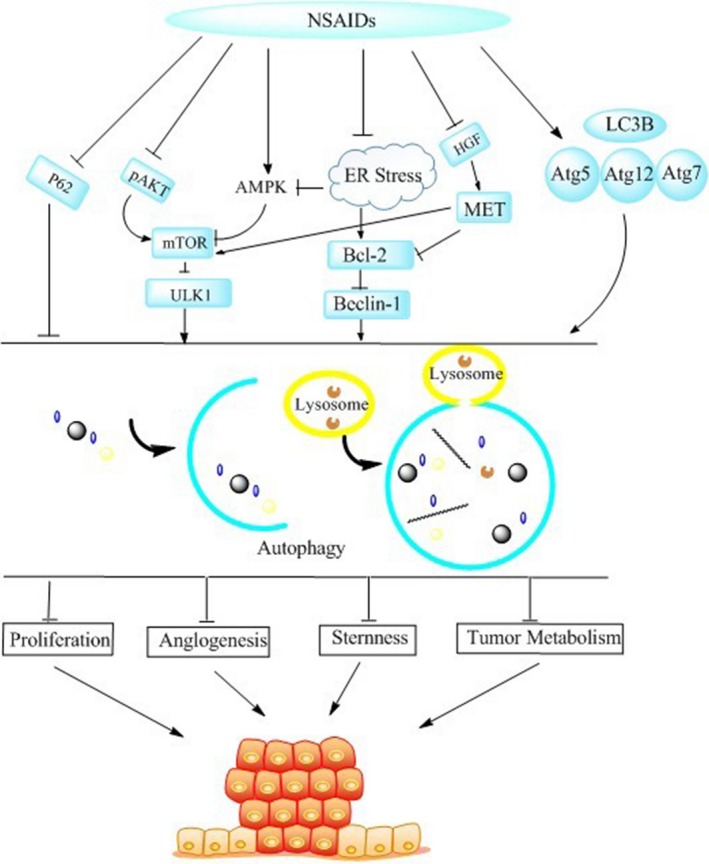
NSAIDs significantly induce autophagy. The most important pathway is mTOR signaling pathway, including PI3K/AKT and AMPK/mTOR. Similarly, NSAIDs also regulate directly the target genes, such as LC3B, P62, P53, Atg5, Atg12, and HIF‐1, the latest Discovered.

Nonsteroidal anti‐inflammatory drugs (NSAIDs) are a structurally diverse group of drugs that are widely used to treat pain, inflammation, and fever, including acetylsalicylic acid, celecoxib, and acetaminophen. In 2016, the US Preventive Services Task Force recommended low‐dose aspirin for preventing colorectal cancer (CRC) in patients without a bleeding tendency 7. More recently, several novel studies have examined NSAIDs and cancer 8, and some researchers found that NSAIDs are closely related to autophagy, especially in hepatocellular carcinoma (HCC) 9, glioblastoma 10, neuroblastoma 11, acute leukemia 12, lung adenocarcinoma 13, oral cancer 14, breast cancer 15, ovarian cancer, colon cancer 16, bladder cancer^17^, and gastric cancer 18. In this article, we will review the mechanisms underlying the actions of NSAIDs in autophagy (Fig. 1; Tables 1 and 2). The use of NSAIDs in combination with chemotherapeutic drugs appears to be a promising approach for the treatment of drug‐resistant tumors that deserves further investigation.

**Table 1:** NSAIDs that induce tumor suppressing autophagy

NSAIDs/chemotherapeutics	Mechanism of action	Cancer cell line	Animal models	Regulation of autophagy	Author (Refs.)
Celecoxib	Conversion of LC3 from LC3I to LC3II↑cleavage of caspases‐8,‐9, ‐3↑Bid↓	HT‐29HCT116colorectal cancer cells		Enhance	Shengbing et al. 6
Celecoxib	ERS↑LC3‐II	MDA‐MB‐231MDA‐MB‐468Hs578t T47D JCBreast cancer cells	MCF‐7MDA‐MB‐468	Enhance	Simmy et al. 15
Celecoxib/*γ*‐irradiation	LC3‐II ↑GADD153/CHOP GRP78/BiP ↑P21Waf1 P27Kip1↑ P53↓	U87MGU251MGGL261malignant glioma cells		Enhance	Kenshi et al. 36
Celecoxib/CPT‐11	mTOR↑LC3‐II↑		TNB9TS‐N‐2nu	Enhance	Setsuko et al. 11
Celecoxib/MDR	HGF/MET↓Bcl‐2 mTOR↓TGFb1,p16INK4b, P21Cip1 P27Kip1↑	PLC/PRF/5P5		Enhance	Roberto et al. 66
Celecoxib	p53 p21↑LC3‐II↑DNA synthesis↓	U87MGU87MG‐E6LN229		Enhance	Khong et al. 37
Celecoxib/Sildenafil	Beclin1↓Atg5↓	GBM5/6/12/14		Enhance	Laurence et al. 85
Celecoxib	P‐Akt↓ caspase‐8 ‐9↑procaspase‐8 ‐9↓	SGC‐7901		Enhance	MIN et al. 18
Celecoxib	LC3 II↑ LC3‐I↑P62↓ JNK↑	PC3		Enhance	Xin et al. 86
Sodium Salicylate	GD‐induced CuZnSOD↓HMGB↓ ROS production↓	A549		Enhance	Sung‐chul et al. 87
Aspirin	mTOR↓ AKT↓AMPK↑phosphorylation of S6K1,S6↓	RKOSW480HCT116		Enhance	Farhat et al. 62
Aspirin	p‐mTOR↓HIF‐1*α*↓VEGF‐A↓ULK1↑ LC3A↑		H22S180	Enhance	Qianqian et al. 65
Sulfasalazine	LC3‐II↑Atg5‐12↑p‐Akt↓ p‐ERK↑	HSC‐4		Enhance	Hye‐Yeon et al. 13
Sulfasalazine	NF‐*κ*B↓P62↓	U251		Enhance	Jing et al. 88
Piroxicam/carboplatin	Vacuoles↑	T245637		Enhance	JéSSICA et al. 89
Indomethacin	Smad7↓	RGM1		Enhance	Ho‐Jae et al. 10
OSU‐03012	ERK1/2↓Atg5↑ROS accumulation↑cleaved LC3↑	Huh7Hep3BHepG2	Huh7	Enhance	Gao et al. 28
OSU‐03012	AKT↓ERK1/2↓	GBM12		Enhance	Laurence et al. 85

**Table 2:** NSAIDs that induce cytoprotective autophagy

NSAIDs/chemotherapeutics	Mechanism of action	Cancer cell line	Autophagy inhibitor	Regulation of autophagy	Author (Refs.)
Celecoxib/ imatinib	Lysosome function↓ p62↑	KBM5KBM5‐T315I		Inhibit	Ying et al. 7
Celecoxib	Lysosome function↓LC3‐II↓ p62↑	HL‐60		Inhibit	Ying et al. 12
Celecoxib	Atg12‐Atg5 conjugate↑LC3B↑	NTUB1T24	3‐methyladenine Bafilomycin A1ATG7 siRNA	Enhance	Kuo‐How et al. 5
Celecoxib	LC3‐II↑P62↓	HT‐29 SW480HCT116	3‐methyladenineAtg8/LC3BsiRNA Vps34 siRNA	Enhance	Shengbing et al. 6
Sulindac Sulfide	Cytochrome c↑	HT‐29	3‐methyladenine	Enhance	Bauvy et al. 91
Aspirin	Mcl‐1↓LC3II/I ratios early↑‐later↓p38↑	HO‐8910H1299 A549HCT‐116 HT‐29	3‐methyladenineBafilomycin A1	Enhance (short‐term)inhibit(long‐term)	Zhang et al. 16
Meloxicam	Beclin 1, LC 3‐II↑p‐AKT↓	HepG2Bel‐7402 Huh‐7	3‐methyladeninechloroquine	Enhance	Xiaofeng et al. 85
Meloxicam	GRP78↑Beclin‐1, Atg5,Atg7 LC3↑P62↓	HepG2Bel‐7402 cells	3‐methyladenineAtg5 siRNA	Enhance	Jingtao et al. 85

## Bcl‐2 family: regulation of autophagy

**Figure 2:**
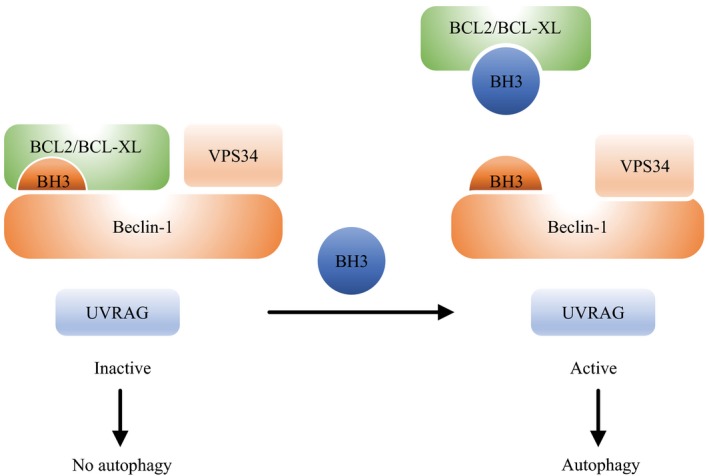
Among the initial steps of vesicle nucleation is the activation of mammalian Vps34, a class III phosphatidylinositol 3‐kinase (PI3K), to generate phosphatidylinositol‐3‐phosphate. Vps34 activation depends on the formation of a multiprotein complex of beclin‐1, UVRAG, and BH3^93^.

Bcl‐2 family proteins contain Bcl‐2 homology (BH) domains, and are divided into three types: antiapoptotic proteins (e.g., Bcl‐2, Mcl‐1, and Bcl‐w), proapoptotic proteins (e.g., Bax and Bak), and BH3‐only proteins (e.g., Beclin‐l and Bid). BH3‐only proteins play a role in promoting apoptosis by directly activating or antagonizing other BH‐containing proteins. The Bcl‐2 family of proteins regulates the mitochondrial membrane via dimerization, and their effects on autophagy can be regulated bidirectionally. Bcl‐2 family protein regulate autophagy by blocking calcium release from the endoplasmic reticulum, thereby inactivating calmodulin‐dependent kinase and adenosine monophosphate‐activated protein kinase (AMPK) and inhibiting mammalian target of rapamycin (mTOR) activity, which promotes autophagy 19. Beclin‐1 and Bcl‐2/Bcl‐xL interact via the Bcl‐2 homology (BH3) domain in Beclin‐1, and dissociation of Beclin‐1 from Bcl‐2/Bcl‐xL leads to increased autophagy 20. Beclin‐1 has been shown to be involved in the formation of autophagic vacuoles (Fig. 2), and other proapoptotic signals, such as Bad and Bax proteins, also activate autophagy 21. Beclin‐1, phosphoinositide 3‐kinase (PI3K), and Atg14 form a heterotrimer and bind the autophagy‐related proteins. In the presence of Atg4, the microtubule‐associated protein 1A/1B‐light chain 3 (LC3) precursor is cleaved into the soluble LC3‐I, which associates with phosphatidyl ethanolamine (PE) to form a LC3‐II‐PE conjugate. This LC3‐II‐PE conjugate is recruited to the autolysosomal membrane.

## Signaling pathways that control autophagy

### Growth factor signaling: mitogen‐activated protein kinase/extracellular signal–activated protein kinases 1 and 2, phosphatidylinositol‐3‐kinase/Akt, and the mammalian target of rapamycin signaling pathway

The conserved mTOR protein complex includes mTORC1 and mTORC2. mTORC1 acts as a nutrient sensor and thus plays an important role in the regulation of autophagy 22. When mTORC1 is activated, autophagy is inhibited. The upstream regulators of mTORC1 are PI3K and Akt, and Akt kinase modulates the stability of the TSC1‐TSC2 protein complex. The TSC1‐TSC2 complex can also receive input from other signaling pathways, such as extracellular signal‐activated protein kinases 1 and 2, and some studies have shown that phosphorylation of TSC2 by Akt or ERK1/2 can dissociate the TSC1‐TSC2 complex, thereby activating mTORC1 23. Under hunger or hypoxia, the PI3K/Akt/mTOR signaling pathway and the Bcl‐2 family of proteins suppress autophagy 24.

### Energy sensing pathway: AMPK positively regulates autophagy

Adenosine 5′‐monophosphate (AMP)‐activated protein kinase (AMPK) is a cellular energy sensor that is activated by hepatic protein kinase B1 (LKB1) when nutrient or energy expenditure [i.e., the adenosine monophosphate (AMP)/adenosine triphosphate (ATP) ratio] is reduced. In contrast to the Akt and ERK1/2 pathways, which activate mTOR 1, activated AMPK can phosphorylate and activate the TSC1‐TSC2 complex, which in turn inhibits formation of the mTORC1 complex 25. AMPK can act as a positive regulator of autophagy by directly activating ULK1, a serine/threonine‐protein kinase, through phosphorylation of ULK1 at Ser317 and Ser777. AMPK also inhibits the activity of mTORC1 by directly phosphorylating its receptor, a regulatory‐associated protein of mTOR 26.

### Autophagy, a double‐edged sword in tumor progression

Autophagy has dual functions; in normal tissues, autophagy is a physiological process that can clear away damaged organelles to maintain cell metabolism at steady‐state. However, rising autophagy levels can cause “autophagic cell death.”^27^ Autophagic cell death, also known as type II programmed cell death, occurs in response to various anticancer therapies 28. Inducing autophagy has also been shown to improve the outcome of tumor treatments 29, 30, 31, 32, 33. However, when autophagy is cytoprotective, inhibition of autophagy has been shown to enhance the efficacy of antitumor treatment 34, 35, 36. Certain pathways of programmed cell death have been shown to be inhibited either in the presence of autophagic inhibitors or when the expression of the autophagy‐specific genes (ATGs) that regulate autophagy is reduced 37, 38. Recently Lu et al. 39 suggested that the balance between proautophagic and antiautophagic activity in the cellular microenvironment determines whether cancer cells die an autophagic death, remain dormant, or exit dormancy.

## Autophagy: novel applications of NSAIDs

### Anticancer effects and autophagy regulation of NSAIDs

A group of drugs that share similar mechanisms of action and are clearly distinct from other groups of drugs used for the treatment of inflammation, are collectively named “NSAIDs.”^38^ These drugs are inhibitors of the enzyme cyclooxygenase (COX) and affect the production of prostaglandin (PG) signaling molecules. There are two isoforms of COX; COX‐1, which is involved in the production of PGs in the gastrointestinal tract under basal conditions, and COX‐2, which is activated by growth factors, mitogens, and tumor promoters 28. COX‐2 is the rate‐limiting enzyme in prostaglandin synthesis. High expression of COX‐2 can promote the synthesis of prostaglandin E2 (PGE2), which induces cell proliferation and stimulates the expression of Bcl‐2 40. COX‐2 overexpression is closely related to the occurrence and development of tumors 41. Compelling data from large studies indicate that aspirin and other NSAIDs are associated with a decreased risk of colorectal, lung, and other carcinomas 27, 39, 40. It was reported that prostaglandin E2 (PGE2) may modulate various immune responses by inhibiting apoptosis and autophagy, and stimulate cancer cell proliferation 37, 42. However, the underlying mechanism is unclear. One study investigated the relationship between prostaglandin E2 (PGE2) production and autophagic cell death in human patellar tendon fibroblasts (HPTFs) in vitro 41. Since the 1970s, numerous large epidemiologic studies and meta‐analyses have strongly supported a protective association between the ingestion of aspirin/other NSAIDs and adenocarcinomas 38, 43, 44. A meta‐analysis of 11 randomized clinical trials by Chen et al. 45 reported that celecoxib was beneficial for the treatment of various types of advanced cancer. Specifically, NSAIDs have various antitumor effects related to apoptosis, autophagy, and tumor immunity. However, numerous questions remain unanswered. For example, which patients can be effectively treated with NSAIDs? How can efficacy be improved? What roles could NSAIDs play in chemotherapy in combination therapy or as an adjunct? Some studies have shown that increased inflammation is associated with an increased risk of cancer, and that obesity further increases the inflammatory response. For obese people, oral aspirin is more effective for preventing tumors 46. Some studies have shown that the effect of aspirin on tumor prevention is dose‐dependent 47. However, another study found that 100 mg/day is the best dose of aspirin for reducing the incidence of adverse reactions, such as bleeding 48. Accumulating evidence suggests that the use of low‐dose aspirin can prevent and reduce the risk of cancer 49. The combination of chemotherapy drugs with NSAIDs, which reduce the side effects of chemotherapy, has been shown to be very effective. Some research has reported that combination treatment with imatinib and celecoxib had an additive synergistic cytotoxicity effect in imatinib‐resistant chronic myeloid leukemia cells (*Q* > 0.85) 9. Celecoxib, acting as an alternative autophagy inhibitor, might serve as a new tool to enhance the antitumor activity of therapeutic agents, especially those that induce cytoprotective autophagy. However, these issues require further study.

### NSAID‐induced autophagy has diverse anticancer effects by regulating Beclin‐1, LC3‐II, p62, and Atg5‐12

The autophagy‐related gene BECN1 encodes Beclin‐1, which is an important regulator of autophagy. BECN1 is located on 17q21 and it encodes a 450‐amino acid polypeptide 8. In many tumors, research has shown that Beclin‐1 could be used as a marker of tumor suppressor loss during tumor development 29, 30. The autophagosome‐associated protein light chain 3 (LC3), also called autophagy‐related gene 8 (Atg8), is a marker of autophagy that exists in two forms, LC3‐I and LC3‐II, that are produced posttranslationally. At the onset of the autophagy cascade, cytosolic LC3‐I is first converted to membrane‐bound LC3‐II. This conversion is triggered by celecoxib, and LC3‐II is thought to be present on autophagosome membranes. Zhong et al. 50 reported that meloxicam induced cell cytoprotective autophagy by upregulating Beclin‐1 and LC3‐II in HCC cells. Specific inhibition of autophagy by 3‐methyladenine and chloroquine (CQ) enhanced the proapoptotic effects of meloxicam by upregulating the expression of Bax 50. However, Suzuki et al. 51 showed that LC3‐II expression is enhanced by celecoxib treatment in hypoxic glioblastoma cells when compared to the expression levels in normoxic glioblastoma cells. Autophagic cell death also occurred. The same results were obtained in various tumor cell lines, such as TNUB1 urothelial carcinoma cells 56, U87MG glioblastoma cells 10 and MCF‐7 breast cancer cells 15 in vivo and in vitro. Not only did meloxicam and celecoxib notably increase the levels of Beclin‐1 and LC3‐II/LC3‐I but they also significantly reduced p62 levels. P62 is degraded during autophagy and can be used as a marker of autophagic flux 52. P62 also accumulates in autophagy‐deficient cells 52. Huang and Sinicrope 67 hypothesized that cotreatment of HCT116 cells with celecoxib and a small‐molecule Bcl‐2/Bcl‐xL antagonist (ABT‐737) reduced the level of p62 protein compared to reduction induced by either drug alone, and increased the conversion levels of autophagosome‐associated protein LC3. The adaptor protein p62 is a well‐known marker of autophagy and participates in crosstalk with important signaling pathways, including the nuclear factor‐*κ*B (NF‐*κ*B) signaling pathway 26, 53. Substantial research has confirmed that the adaptor protein p62 participates in the regulation of NF‐*κ*B signaling, although the mechanism is not fully understood 54. However, Yu et al. 55 showed that p62 is involved in cisplatin‐resistance in ovarian cancer cells through autophagy.

It is interesting to note that celecoxib had the opposite effect on autophagy in chronic myelogenous leukemia (CML) than in solid tumors, as previously reported 9. Ying et al. published that celecoxib prevents autophagic flux by inhibiting lysosome function at its late stages and enhances the cytotoxicity of imatinib in imatinib‐resistant KBM5‐T315I cells. The expression levels of p62 protein were increased when cells were treated with celecoxib. The same results were obtained using cells from patients with chronic myelogenous leukemia (CML). The main finding of this study, that celecoxib acts as an autophagy suppresser in HL‐60 cells, will help to expand the applications of celecoxib to different forms of leukemia. Another group reported that celecoxib suppresses autophagy in HL‐60 cells by altering the acidic environment of the lysosomes, and that celecoxib induces cell apoptosis and necrosis in HL‐60 cells 12. Since celecoxib can act as an inhibitor of autophagy, it could be used as a new tool for enhancing the antitumor activity of therapeutic agents, especially agents that induce cytoprotective autophagy in acute myeloid leukemia (AML) cells (Fig. 1).

There are more than 20 proteins involved in the autophagy pathway downstream of mTOR. Research has shown that the levels of the conjugated Atg5‐Atg12 protein increase significantly after treatment with celecoxib, meloxicam, or OSU‐03012 5, 41, 56. OSU‐03012 is a derivative of celecoxib, a COX‐2 inhibitor, with anticancer activity that has been shown to induce the death of various types of cancer cells 57. The overexpression of autophagy‐related gene5 (Atg5) not only promotes autophagy but also promotes apoptosis 58. The proapoptotic effect of autophagy‐related gene5 (Atg5) is related to its regulation of Bcl‐xL (i.e., through the release of cytochrome C and the activation of calpain and caspases) 59.

### Regulation of NSAID toxicity by endoplasmic reticulum stress proteins

The endoplasmic reticulum (ER) is a cellular stress response system 60, 61 that has two main functions: (1) under moderate stress, this system defends against and neutralizes the insult to restore homeostasis. A critical component of this protective response is glucose‐regulated protein 78 (GRP78), a chaperone and calcium‐binding protein whose expression is increased in response to stress 62. (2) Under excessive stress, the ER system switches to its proapoptotic mode and initiates apoptosis. A central player in this process is CHOP (also known as GADD153), a transcription factor that alters the transcriptional profile of cells and triggers the proapoptotic pathway 63. ER stress has been previously reported to induce autophagy 64, 65. When misfolded proteins accumulate in the ER under stress, the stress activates the unfolded protein response, which augments the expression of proteins involved in the recovery process. As previously reported, celecoxib is a known inducer of ER stress. Induction of autophagy after celecoxib treatment may be associated with the cellular response to ER stress 66. Autophagy is known to play an important role in coping with multiple forms of cellular stress, including nutrient or growth factor deprivation, hypoxia, or damaged organelles 65, 67, 68, 69. Celecoxib can induce the activation of stress‐related molecules, such as phospho‐eIF2a, activating transcription factor 4 (ATF‐4), phosphor‐stress‐activated protein kinase/c‐Jun NH(2)‐terminal kinase (SAPK/JNK), and phospho‐c‐Jun. The induction of autophagy after celecoxib treatment may be associated with these stress responses.

Some researchers reported that the Bip/GRP78 signaling pathway activates NSAID‐induced autophagy 64, 65. Celecoxib is a calcium pump inhibitor that has potential therapeutic effects. However, Johnson et al. 65 reported that celecoxib could increase intracellular calcium ion levels, and induce the ER‐mediated effects of GRP78 and CHOP. Under normoxic conditions, the levels of the ER stress marker GRP78 increased as the concentration of celecoxib increased in all tested cell lines over the concentration range examined 65. In addition, GRP78, CHOP, and LC3‐II expression levels in the U87MG and U251MG cell lines are comparable after treatment with celecoxib with or without radiation, particularly under hypoxic conditions 65.

### NSAIDs activate autophagy by blocking mTOR signaling

**Figure 3:**
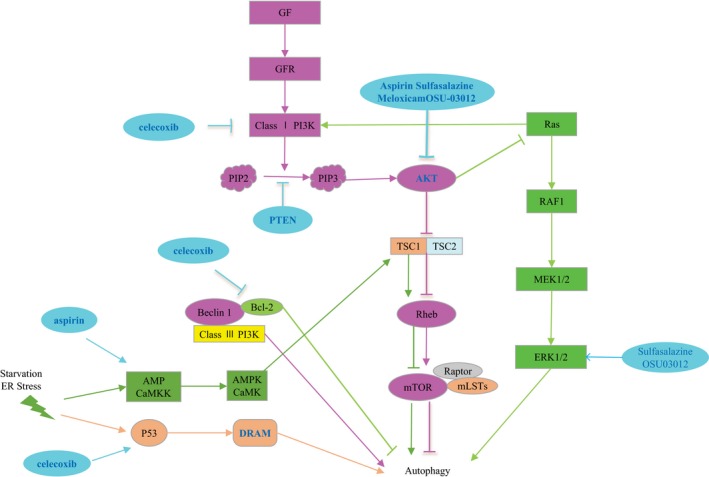
Upstream cell signaling pathway of autophagy. Fuchsia: growth factor activates PI3K/AKT/mTOR signaling pathway; dark green: starvation and endoplasmic reticulum stress promote autophagy through AMPK/CaMKK signaling pathway; dark yellow: P53/DRAM signaling pathway; pale green: Ras signaling pathway can not only promote autophagy but also inhibit autophagy; and light blue: various NSAIDs modulate different signaling pathways to promote autophagy.

Multiple signal transduction mechanisms are known to regulate autophagy; however, the mTOR pathway is the most important pathway in the onset of autophagy 29. mTOR is mainly activated by the PI3K/Akt/mTOR signaling pathway, and it is a central checkpoint that negatively regulates autophagy. In contrast, anticancer drugs that inhibit the PI3K/Akt/mTOR axis augment autophagy progression. Additionally, adenosine monophosphate‐activated protein kinase (AMPK) is a pivotal cellular energy sensor that recognizes cellular ATP starvation; its downstream targets include the negative regulator of mTOR 67. AMPK‐mediated inhibition of mTOR phosphorylation and activity induces autophagic responses in a variety of different cell types 68. For example, Din et al. 72 found that aspirin activates AMPK and reduces mTOR signaling in CRC cells by inhibiting the mTOR effectors S6K1 and 4E‐BP1. Aspirin and metformin (an activator of AMPK) promote the inhibition of mTOR and Akt, as well as autophagy in colorectal cancer cells 69, 70. As described previously, Zhao et al. 71 proposed that aspirin may inhibit angiogenesis and induce autophagy by inhibiting the mTOR signaling pathways in murine hepatocarcinoma and sarcoma models; the expression levels of p‐mTOR, hypoxia‐inducible factor 1‐alpha (HIF‐1*α*), and vascular endothelial growth factor‐A (VEGF‐A) were decreased, while the expression levels of Unc‐51‐like kinase (ULK1) and LC3A were increased following treatment of the H22 and S180 cell lines with aspirin and everolimus (an inhibitor of mTOR). Other studies have shown that there is no significant change in the messenger RNA (mRNA) levels of Akt following treatment with celecoxib 18. However, phospho‐Akt levels decreased in a time‐ and dose‐dependent manner 18. Celecoxib regulates autophagy via the PI3K/Akt signaling pathway in SGC‐7901 gastric cancer cells. Researchers have shown that different NSAIDs have the same effect as autophagy‐inducing drugs, such as meloxicam 50, sodium salicylate 71, and sulfasalazine 14. Mazzanti et al. 72 reported similar results; exposure of cells to a low concentration of celecoxib downregulates mTOR expression and causes G1 cell‐cycle arrest with autophagy in the human multidrug‐resistant overexpressing HCC cell line PLC/PRF/5, whereas higher concentrations of celecoxib trigger apoptosis. Furthermore, Setsuko et al. 62 found that mTOR gene expression was downregulated in drug‐resistant tumors when treated with a low dose of irinotecan (CPT‐11) in combination with celecoxib for 20 consecutive days. However, in chemosensitive neuroblastoma xenografts, mTOR gene expression was significantly upregulated compared to that in untreated controls. Hence, we can speculate that the effect of NSAIDs in tumor cells switches between autophagy and apoptosis, depending on the concentration and the time of administration. Low concentrations of NSAIDs can induce autophagy at early stages, whereas higher concentrations of NSAIDs can inhibit autophagy and induce apoptosis at later stages (Fig. 3). More research is required to determine whether drug resistance has an effect on the mTOR pathway, which can affect the progression of autophagy.

### NSAIDs inhibit tumor cell viability through p53‐dependent autophagy

Mutational inactivation of the tumor suppressor gene p53 is frequently detected in human tumors, and p53 mutation/inactivation is reported in 63–65% of high‐grade gliomas 74. The presence of DNA damage initiates a signaling cascade, p53 activation (by phosphorylation at Ser15 and Ser20), and subsequent transcriptional activation by p53 75. Genotoxic stress caused by DNA‐damaging agents also induces p53‐dependent autophagy 76, 77, 78. Some reports have suggested that celecoxib inhibits glioma proliferation through p53‐dependent induction of autophagy, but not apoptosis 10. Celecoxib did not induce significant levels of autophagy in U87MGPFT, U87MG‐E6, or U373MG human glioblastoma cell lines, which lack functional p53. The mechanisms of p53‐dependent autophagy are not fully understood, but are thought to involve both transcription‐independent functions (e.g., activation of the nutrient energy sensor AMPK) and transcription‐dependent functions (e.g., upregulation of the mTOR inhibitors phosphatase and tensin homolog (PTEN) and TSC1 or the p53‐regulated autophagy and cell death gene DRAM) 78 (Fig. 3). The antiproliferative mechanism of COX‐2 inhibitors is unclear, and how they induce autophagy in tumors is unknown. Liu et al. 18, 79 hypothesized that crosstalk between p53 80, PI3K/Akt 81, and Bcl‐2‐beclin‐1 pathways was involved in the autophagy/apoptosis switch 82. Thus, the role of p53 in celecoxib‐induced autophagy must be clarified.

### NSAID‐induced autophagy enhances radiosensitivity and the cytotoxicity of chemotherapy drugs

Many studies have shown that antitumor therapy, including radiotherapy and chemotherapy, can induce apoptosis, but it can also induce autophagy. Under these conditions, autophagy is often a protective mechanism against various treatments to inhibit apoptosis of tumor cells, which are often drug‐ and radiation‐resistant. Therefore, if autophagy can be inhibited in tumor cells during antitumor therapy, the apoptosis signaling pathway will need to compensate by initiating the programmed cell death of tumor cells, which could improve the efficacy of antitumor treatments.

**Figure 4:**
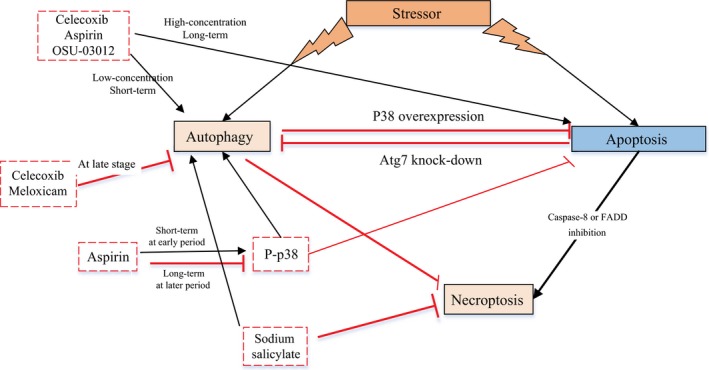
Interaction between different types of programmed cell death and NSAIDs. At late stage and longer term, NSAIDs (e.g., celecoxib, meloxicam, and aspirin) switch from autophagy to apoptosis. At early period and shorter term, they switch from apoptosis to autophagy.

The role of autophagy in tumor resistance has also become the focus of recent research 83. In a variety of tumor models, autophagy can remove abnormal proteins, organelles, and reactive oxygen species from within the cell, which plays a role in its drug resistance. When apoptosis in tumor cells was induced by chemotherapeutic drugs, autophagic activity was increased, which led to drug resistance; however, the combination of chemotherapeutic drugs with an autophagy inhibitor could reduce this drug resistance 73. The following is a review of autophagy inhibitors, which are frequently used for protective autophagy: 3‐methyladenine (3‐MA), which inhibits class III PI3K activity; chloroquine (CQ) and hydroxychloroquine (HCQ), which inhibit the fusion of autophagic bodies and lysosomes by altering lysosomal pH values; bafilomycin A1, which inhibits the activity of vacuolar‐ATPase; and small interfering RNA (siRNA) which inhibits the expression of autophagy‐related gene (Atg) (Table 2). However, under certain conditions, autophagy can also be involved in apoptosis induction. In fact, apoptosis may become the major mode of cell death. High mobility group protein B1 (HMGB1) is a protein released by tumor cells in response to the cytotoxic effects of chemotherapeutic agents. HMGB1 activates autophagy through the PI3K/Akt signaling pathway to enhance drug resistance. The ability of salicylate to prevent necrotic death may contribute to its anti‐inflammatory action and may suppress tumor development by switching the cell death pathway from tumor‐promoting necrotic cell death to tumor‐suppressive autophagic cell death. Switching the method of cell death may provide a new strategy for the development of antitumor therapies 74. Other studies suggest that P‐glycoprotein (P‐gp) expression and the activity of the hepatocyte growth factor (HGF)/MET autocrine loop are correlated in HCC cells. HGF/MET autocrine loop activity can lead to the overexpression of Bcl‐2 and mTOR, which inhibit the translation initiation factor eIF2 alpha, making the tumor cells resistant to both autophagy and apoptosis 73. Celecoxib inhibits the expression of the P‐gp protein in the loop, thereby reversing resistance. Kaneko et al. found that celecoxib could alleviate the negative effect of imatinib on autophagy by upregulating p62 expression 9. Sensitivity to chemotherapy in patients significantly improved, and combination treatment with imatinib and celecoxib had synergistic cytotoxicity effects in imatinib‐resistant cells 7. Inhibition of autophagy has been shown to enhance antitumor drug efficacy. Concurrently, some researchers proposed that celecoxib enhances the radiosensitivity of hypoxic glioblastoma cells by inducing strong ER stress signals 51. Celecoxib is a promising radio‐sensitizing drug for clinical use in patients with glioblastoma. However, some researchers have expressed different views. Researchers have found that celecoxib enhances the cytotoxicity of imatinib in imatinib‐resistant KBM5‐T315I CML cell lines 9. The underlying mechanism may be related to imatinib‐induced autophagy, which could be reversed by celecoxib. Zhang et al. 16, 35 found that long‐term combination treatment with aspirin and a small‐molecule Bcl‐2/Bcl‐xL antagonist (ABT‐737) could synergistically induce apoptosis both in A549 and H1299 cell lines. Meanwhile, combination treatment with short‐term aspirin and the small‐molecule Bcl‐2/Bcl‐xL antagonist (ABT‐737) caused a greater autophagic response than that to either drug alone, and autophagy switched from a cytoprotective signal to a death‐promoting signal 16. P38 acts as the cellular switch between these two different cell death pathways (i.e., autophagy and apoptosis) induced by cotreatment with aspirin and ABT‐737 16. Hence, the dose and duration of administration can also be two factors that alter the sensitization effects of NSAIDs (Fig. 4). The sensitivity of tumor cells to celecoxib‐induced cellular autophagy is likely to be concentration‐ and/or tumor‐type dependent. These different effects, in a large part, depend on the type of tumor, disease stage, and method of treatment 30, 84.

### Side effects of NSAIDs and possible mechanisms

Conventional nonselective NSAIDs inhibit both COX enzymes. Inhibition of COX‐1 can lead to acute gastric mucosal lesions 85. Celecoxib is the first selective COX‐2 inhibitor approved for clinical use in the treatment of osteoarthritis and acute pain. It can significantly reduce the incidence of adverse gastrointestinal effects. However, recent studies have shown that long‐term use of selective COX‐2 inhibitors increases the risk of adverse cardiovascular events, especially myocardial infarction 85, 86. This is may be due to the mechanism of NSAIDs by which cardiovascular events are induced, mainly by inhibiting COX‐2 in endothelial cells, thereby inhibiting the production of prostacyclin I (prostacyclin, PGI2). PGI2 can dilate blood vessels and inhibit platelet aggregation. A reduction in PGI2 production increases the risk of vascular injury and tendency for thrombus. To balance the benefits of aspirin and the risk of bleeding, patients should be evaluated, by analyzing them for PIK3CA mutations. To use NSAIDs to effectively prevent tumor formation and progression, we can evaluate COX‐2 expression and gene polymorphisms. The proposed anticancer mechanism for NSAIDs is as follows. NSAID_S_ exert anticancer effects by reducing the frequency of gene mutations, inhibiting prostaglandin peroxidase synthase‐2 activity, inhibiting inflammation, and regulating autophagy. Although both observational and randomized studies provided convincing evidence for aspirin as an effective chemopreventive agent for CRC 87 it is not recommended for primary prevention of CRC in people at average risk of chronic venous diseases (CVD) because of the potential risks. Aspirin is used for secondary prevention of CRC, particularly in certain subgroups, such as those with wild‐type KRAS tumors 88. Future studies are needed to find the optimal timing, dose, and duration of NSAID use and to identify subgroups of patients with cancer for whom the benefits of aspirin outweigh its risks.

## Summary and future perspectives

NSAIDs have been used as anti‐inflammatory, analgesic, and antithrombotic drugs 69. Hence, the safety and side effect profiles of these drugs are well understood. These drugs are good clinical candidates for further exploration of their underlying mechanisms of chemoprevention in cancer 21. The antitumor effects of NSAIDs are related to their autophagy‐modulating effects: either activation or inhibition of autophagy. Thus, it is important to determine the fate of tumor cells treated with NSAIDs. The net effect of autophagy in cancer may depend on the type of tumor, stage of tumorigenesis, tumor microenvironment, as well as genetic and epigenetic factors. One of the most effective treatment methods is to combine lower dose and longer term NSAID administration with chemotherapeutic drugs. NSAIDs are commonly used antipyretic and analgesic drug. With further research, NSAIDs could play an important role in combating tumor progression, especially with the discovery of new mechanisms and the development of molecular biology techniques to study autophagy. Understanding the effects of NSAIDs and their antitumor effects at the molecular and cellular levels will improve our understanding of tumor pathogenesis and uncover potential anticancer targets.

## Conflict of Interest

All the authors declare that they have no conflict of interest.

## Ethical approval

This article does not contain any studies with human participants performed by any of the authors.
